# Autoimmune bullous diseases: pathogenesis and clinical management

**DOI:** 10.1186/s43556-025-00272-9

**Published:** 2025-05-15

**Authors:** Xun Feng, Huaping Zheng, Mi Wang, Yiyi Wang, Xingli Zhou, Xiwen Zhang, Jishu Li, Yue Xiao, Mintong Wei, Xiaoguang Li, Takashi Hashimoto, Jingyi Li, Wei Li

**Affiliations:** 1 Department of Dermatology & Venerology, Rare Diseases Center, West China Hospital, Sichuan University, Chengdu, Sichuan China; 2 Department of Respiratory and Critical Care Medicine, Center for High Altitude Medicine, Institutes for Systems Genetics, West China Hospital, Sichuan University, Chengdu, 610000 China; 3 Daqing Oilfield General Hospital, Daqing, China; 4 Department of Dermatology, Graduate School of Medicine, Osaka Metropolitan University, Osaka, Japan

**Keywords:** Autoimmune bullous diseases, Autoantibody, Immunogenic domain, Pathogenesis, Targeted therapy, Inflammation

## Abstract

Autoimmune bullous diseases (AIBDs) represent a heterogeneous group of immune-mediated disorders characterized by life-threatening blistering of the skin and mucous membranes. This Review synthesizes current understanding of AIBD pathogenesis, clinical phenotypes, diagnostic approaches, and therapeutic strategies, emphasizing recent advancements and translational opportunities. At the core of AIBDs is autoantibody-mediated disruption of structural proteins in the epidermis or basement membrane zone, particularly at desmosomal and hemidesmosomal junctions. Key subtypes, including pemphigus, paraneoplastic pemphigus, pemphigoid, and IgA-related diseases, are distinguished by their target antigens, clinical manifestations, and immunopathological profiles. Diagnostic workflows rely on direct immunofluorescence, and serological assays, yet subtype differentiation remains challenging due to overlapping features. Traditional therapies, such as systemic corticosteroids and immunosuppressants, have improved outcomes but are limited by toxicity. Recent breakthroughs highlight targeted interventions, including B-cell depletion with rituximab, cytokine modulation via dupilumab, and JAK inhibitors for inflammatory pathways. Innovative strategies like chimeric autoantibody receptor T-cell (CAART) therapy further address refractory cases by eliminating autoreactive B cells. Additionally, the Review underscores the emerging role of inflammation-driven mechanisms and the necessity of multidisciplinary care, given AIBDs’ associations with malignancies, autoimmune comorbidities. Despite progress, challenges persist in early diagnosis, personalized therapy optimization, and understanding antigen-specific immune responses. Future directions include refining diagnostic biomarkers, exploring novel targets, and developing precision medicine approaches.

## Introduction

Autoimmune Bullous Diseases (AIBDs) are life-threatening diseases characterized by autoantibodies targeting structural proteins in the epidermis or basement membrane (BMZ) of the skin and mucosa [1, 2]. These autoantibodies disrupt epithelial integrity, leading to blister formation and erosions. Clinically, AIBDs manifest as vesicles, bullae, and erythematous lesions on cutaneous and mucosal surfaces. Despite advances in understanding their pathogenesis and treatment, AIBDs remain diagnostically and therapeutically challenging due to their immunopathological complexity and heterogeneous presentations.

The historical comprehension of AIBDs has evolved substantially. Early descriptions by Wichmann and Willan in the late 18 th and early nineteenth centuries referred to chronic bullous diseases as “pemphigus”. Hebra later refined the classification of pemphigus in 1869, and Auspitz identified acantholysis, a histological hallmark, in 1881 [3]. In 1953, Lever introduced the subepidermal blister classification, delineating AIBDs into pemphigus and pemphigoid [4]. A pivotal milestone occurred in 1964 when Beutner and Jordon demonstrated circulating autoantibodies against intercellular antigens using direct and indirect immunofluorescence, establishing the autoimmune nature of these disorders [5].

**Fig. 1:**
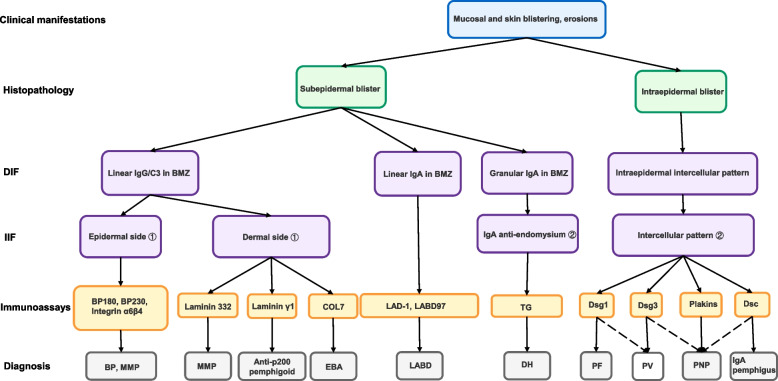
Diagnostic flow chart for autoimmune bullous diseases. Diagnosing autoimmune bullous diseases requires a combination of clinical manifestations, histopathology, immunofluorescence, and detection of circulating antibodies. Abbreviations: DIF direct immunofluorescence, BMZ basement membrane zone, IIF indirect immunofluorescence, COL 7 Type VII collagen, Dsg1 Desmoglein1, Dsg3 Desmoglein3, Dsc desmocollin, PF pemphigus foliaceous, PV pemphigus vulgaris, PNP paraneoplastic pemphigus, BP bullous pemphigoid, LAD linear IgA bullous dermatosis, MMP mucous membrane pemphigoid, EBA epidermolysis bullosa acquisita, DH dermatitis herpetiformis

AIBDs are broadly categorized into two groups: (1) pemphigus disorders (e.g., pemphigus vulgaris, pemphigus foliaceus), characterized by intraepidermal blisters due to autoantibodies against desmosomal proteins, and (2) pemphigoid disorders (e.g., bullous pemphigoid, mucous membrane pemphigoid), marked by subepidermal blistering from autoimmunity to BMZ components. Diagnosing AIBDs requires a multimodal approach integrating clinical evaluation, histopathology, immunofluorescence microscopy, and serological autoantibody detection [6–8]. (Fig. 1) Direct immunofluorescence (DIF) remains the diagnostic gold standard [9], while serological assays like enzyme-linked immunosorbent assay (ELISA) confirm autoantibody presence and monitor disease activity [10, 11]. For example, ELISA kits targeting desmoglein 1 (Dsg1), desmoglein 3 (Dsg3), BP180, and BP230 antibodies have revolutionized diagnostic precision and early detection [12–15]. Recent research has expanded the repertoire of AIBD-associated autoantigens, revealing distinct clinical phenotypes linked to specific antibodies. Anti-laminin 332 antibodies correlate with pharyngo-laryngeal and tracheal involvement [16–18], whereas anti-β4 integrin antibodies are associated with ocular manifestations [19–21]. Such heterogeneity underscores the necessity of precise autoantibody profiling to guide diagnosis, monitoring, and personalized therapies.

Building on classification of AIBD, this review systematically examines four key aspects: (1) Genetic and environmental risk factors contributing to AIBD susceptibility; (2) Pathogenic mechanisms, including molecular targets and immune pathways; (3) Diagnostic advancements, with emphasis on immunofluorescence and emerging techniques; and (4) Therapeutic innovations, spanning conventional immunosuppressants to biologics and small-molecule inhibitors. By synthesizing recent advances in autoantibody characterization, epitope mapping, and targeted therapies, this review aims to bridge translational gaps between research discoveries and clinical practice. Special emphasis is placed on multidisciplinary care models to optimize patient outcomes.

## Pemphigus disease

Pemphigus is a group of autoimmune diseases classified into several subtypes, including pemphigus vulgaris (PV), pemphigus foliaceus (PF), pemphigus herpetiformis (PH), and IgA pemphigus, et al [22]. Different subtypes affect different layers of the skin, have different symptoms, and target different antigens. Major autoantigens in the pemphigus group of diseases include desmogleins (Dsgs), desmocollins, and the plaque proteins, such as desmoplakin, periplakin, and envoplakin [23]. While pemphigus disorders primarily involve autoimmunity to desmosomal proteins, a distinct paraneoplastic variant, paraneoplastic pemphigus/paraneoplastic autoimmune multiorgan syndrome (PNP/PAMS), exhibits broader epitope targeting and systemic complications. This section will specifically focus on epidemiology, genetic and environmental factors, pathogenesis, and therapy of PV and PF, while IgA pemphigus will be discussed in IgA-related AIBDs section due to the unique IgA-mediated pathological mechanism.

### Epidemiology, genetic and environmental factors

PV is the most common subtype of pemphigus, accounting for approximately 70% of all cases [22]. The pooled incidence rate of PV is 2.83 per million person-years [24]. PF, the second most common variant, includes both sporadic and endemic cases. The prevalence of PF is approximately 10 per million [25]. Sporadic PF is a rare condition, comprising 20–30% of pemphigus cases, with an incidence rate of less than 1 per million in the United States and Europe [22].

Numerous studies highlight the critical role of genetic factors in pemphigus. Disease-associated autoantibodies are often detected at low levels in healthy first-degree relatives of pemphigus patients [26]. Specific HLA alleles have been identified as risk factors. For example, HLA-DRB1*14:01 and HLA-DRB1*04:06 are associated with PV in Chinese patients, while *HLA-DQB103:03* and HLA-DQB1*03:02 demonstrate significant subtype specificity [27]. Among Ashkenazi Jews, HLA-DRB1*0402 is predominant, whereas HLA-DRB1*1401, HLA-DRB1*1404, and HLA-DQB1*0503 are prevalent in non-Jewish patients of European and Asian descent [28]. In sporadic PF, genetic factors are also significant, with HLA-DRB1*0101 linked to Mexican patients [29] and HLA-DRB1*04 associated with PF in Brazilian, Dutch, French, and Italian populations [30–32]. Endemic PF presents distinct genetic profiles, such as HLA-DRB1*0404, *1402, *1406, and *0102 in Brazilian patients [33], and HLA-DRB1*03 in Tunisian patients [34]. Environmental factors, including medications, infections, stress, diet, immunizations, and sleep, influence pemphigus pathogenesis [35]. Medications linked to pemphigus fall into three categories: thiol drugs, phenols, and non-thiol/non-phenol drugs [36]. Infections, such as *Legionella pneumophila, Staphylococcus aureus, Proteus vulgaris,* and *Pseudomonas aeruginosa*, and viruses from the Herpesviridae family, have been implicated [37, 38]. Stressful life events and diets rich in thiol-containing compounds, such as garlic, onions, red wine, and beer [36, 39], may also trigger PV. Environmental factors are more pronounced in endemic PF, where poor living conditions, outdoor work, insect exposure, and seasonality are identified as risk factors [40–42].

### Pathogenesis of pemphigus

**Fig. 2:**
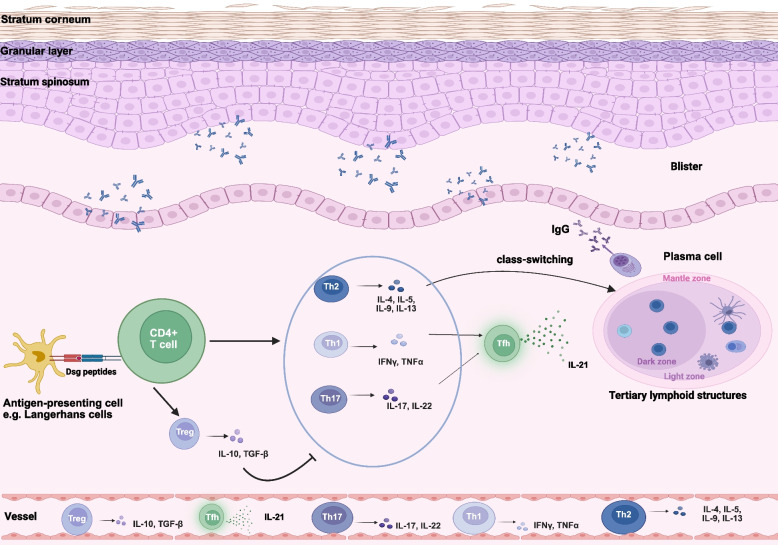
Pathogenesis of pemphigus vulgaris. This figure shows the pathogenesis of pemphigus vulgaris (PV). Autoantibodies binding to autoantigens can directly impair desmosomal function, leading to acantholysis. In the skin, antigen-presenting cells such as Langerhans cells initiate their activation by capturing Dsg peptides and presenting them to CD4 + T cells. Th1 cells enhance the immune response, while Th2 cells regulate the production of pathogenic Dsg-specific IgG antibodies. Th17 cells release IL-17, which drives an inflammatory response, whereas Treg cells inhibit the proliferation of Dsg3-autoreactive T cells and antibody production. Additionally, Tfh cells interact with B cells to facilitate autoantibody production. Some patients develop tertiary lymphoid structures at lesion sites, which may contribute to disease recurrence. Created in https://BioRender.com

Pemphigus disease is a group of autoimmune diseases caused by autoantibodies, in which the autoreactive B cells are activated by autoreactive T cells and differentiate into plasma cells. The autoantibodies produced by these plasma cells bind to desmosomes on the surface of epidermal keratinocytes and cause blisters. Both PV and PF share similar pathological mechanisms, involving the activation of autoreactive T and B cells [43, 44]. B cells contribute by presenting antigens to CD4^+^ T cells and differentiating into plasma cells that produce pathogenic autoantibodies. (Fig. 2).

CD4^+^ T cells play a central role, with imbalances in Th1 and Th2 cells linked to disease progression [45, 46]. PV patients exhibit elevated Th2 cytokines (e.g., IL-4) [47] and decreased Th1 cytokines (e.g., IL-2), with Th2 cells contributing to anti-Dsg3 antibody production [48]. Additionally, Th17 cells are implicated in active disease stages, as their frequency and associated cytokines and chemokines (e.g., IL-17 A, CCL20) are increased in lesions [49–51]. Treg cells, which suppress immune responses [52, 53], are reduced in PV [54, 55], and circulating Tfh cells, which assist B cells, are elevated [46, 56–58]. Regulatory B cells (Bregs), which mitigate excessive inflammation, are diminished in PV, impairing immune regulation [59–61]. Advances in single-cell transcriptomics have revealed key insights into immune cell interactions in pemphigus, for instance, Dsg-specific B cells exhibit genes linked to inflammation and T-cell co-stimulation [62]. The pathogenesis of pemphigus involves increased immune cell populations, enhanced cell communication, and the activation of key pathways such as IL-1α and CXCL13 [63–66], while lesional keratinocytes interact with immune cells via the CCL19-CCR7 axis, promoting autoimmunity [67]. These findings suggest potential therapeutic targets such as inhibiting CXCL13 to address immune dysregulation in PV lesions. Autoantibodies in pemphigus primarily target Dsg1 and Dsg3, with additional antibodies against desmosomal and non-desmosomal proteins contributing to disease complexity [68, 69]. The “multiple hits” hypothesis posits that cumulative antibody interactions disrupt desmosomal function and induce acantholysis [70–72].

### Characteristics of pemphigus

While PV and PF share similar pathological mechanisms driven by autoreactive T and B lymphocytes, they differ significantly in their antigenic targets. In PF, the antigen is restricted to the Dsg1 peptide, whereas, in PV, both Dsg1 and Dsg3 serve as antigens [73, 74]. Dsg1 and Dsg3 are isoforms of desmoglein, a calcium-dependent transmembrane glycoprotein in the cadherin superfamily [75, 76], crucial for keratinocyte cohesion and desmosomal integrity [77]. Autoantibodies in the sera from pemphigus patients predominantly react with the extracellular (EC) domains of Dsg1, particularly its N-terminal adhesive region [78, 79]. Specific epitope mapping identified five regions (aa 86–110, aa 196–220, aa 226–250, aa 326–340, and aa 486–520) within the extracellular domain of Dsg1 [80]. Most conformational epitopes map to the N-terminal 161 amino acids, with a dominant epitope spanning amino acids 26–87 [81]. Further studies revealed that sera from PF patients predominantly react with the EC1 or EC2 domains of Dsg1 [82–86], while some also recognize EC3 to EC5 [83, 84, 87]. Interestingly, Tunisian patients exhibited evidence of epitope spreading from C-terminal domains, contrasting with sporadic PF, where antibodies appear to target N-terminal domains directly [88].

In PV, anti-Dsg3 autoantibodies primarily target the EC domain, with the N-terminal regions being more frequently recognized than the C-terminal domains [89, 90]. Additionally, sera from PH patients have shown reactivity to Dsg3, though the specific domains differ [91]. PH sera, for instance, preferentially bind to the EC4 domain rather than EC1. Four epitope regions (aa 64–78, aa 330–344, aa 375–399, and aa 446–460) have been identified within the EC domain of Dsg3 [80]. Notably, switching between IgG1 and IgG4 subclasses of pathogenic PV monoclonal antibodies does not alter antigen binding or pathogenicity [92]. However, antibodies targeting the EC5 and EC1 domains of Dsg3 may activate signaling pathways that contribute to pathogenic events, including Dsg3 depletion [93].

**Fig. 3:**
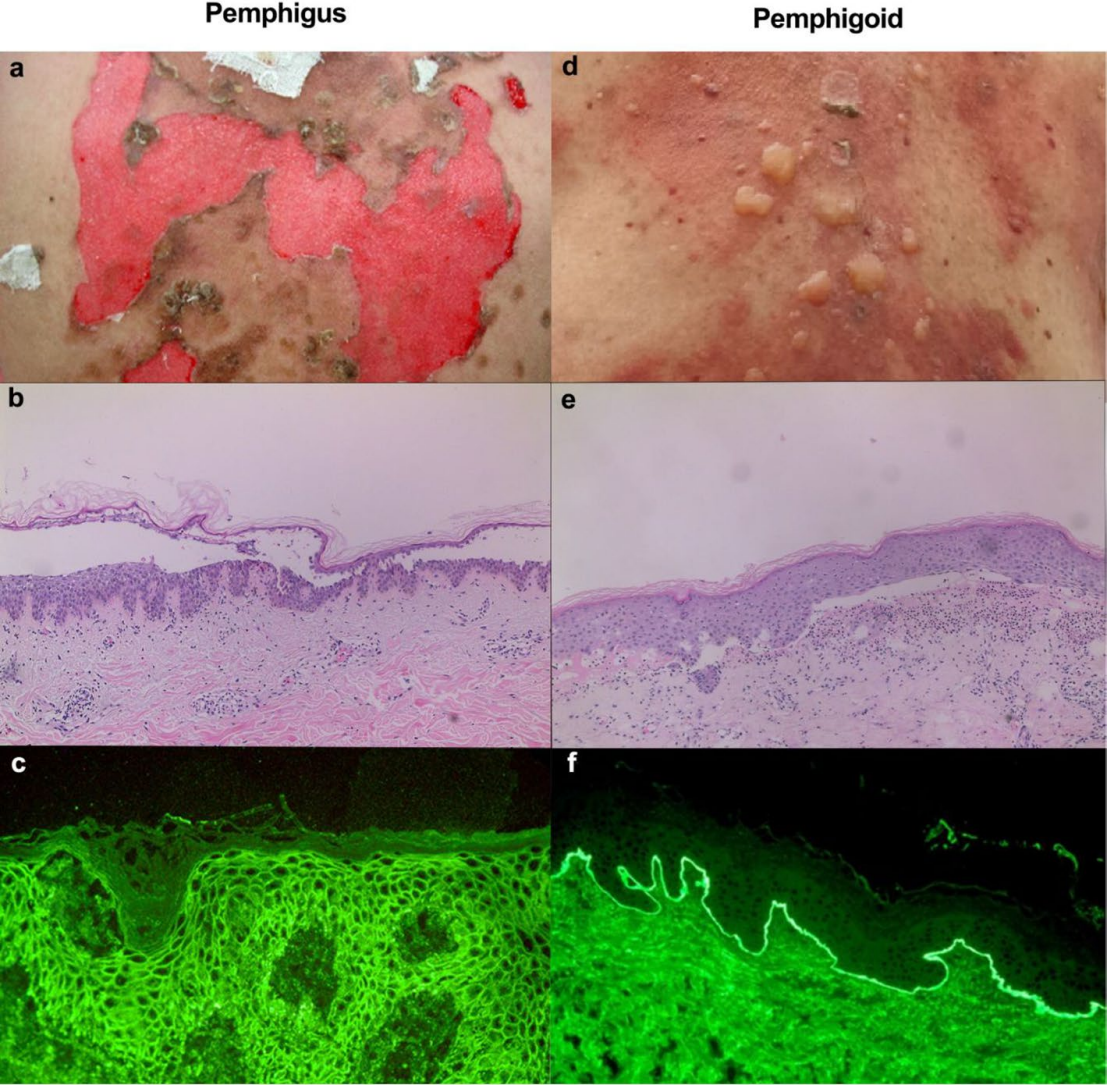
Major clinical and laboratory findings in pemphigus and pemphigoid. Major clinical and laboratory findings in pemphigus and pemphigoid. **a** Extensive erosions with crusts and hyperpigmentation on the back of a female patient with pemphigus vulgaris. **b** Histopathological examination reveals sub-corneal acantholysis and an inflammatory infiltrate. **c** Direct immunofluorescence microscopy analysis of a perilesional skin biopsy shows deposits of IgG with an intercellular pattern in the epidermis. **d** Blisters, and erosions with crusts on an erythematous background in a male patient with bullous pemphigoid. **e** Histopathological examination reveals subepidermal cleavage with an inflammatory infiltrate consisting predominantly of eosinophils and neutrophils. **f** DIF shows IgG with a linear deposition pattern in the basement membrane zone

The distribution of Dsg1 and Dsg3 varies between skin and mucosa, influencing clinical presentations. Dsg1 is abundant in the superficial epidermis, while Dsg3 is concentrated in basal and parabasal layers. Patients with mucosal-dominant PV produce anti-Dsg3 autoantibodies, whereas those with mucocutaneous PV generate both anti-Dsg1 and anti-Dsg3 autoantibodies [94, 95]. This explains the distinct clinical features: PV typically begins with painful oral mucosal lesions that can impair eating and lead to weight loss. Ocular, nasal, laryngeal, esophageal, genital, and anal erosions may occur, followed by flaccid cutaneous bullae, especially in sites of mechanical stress (Fig. 3a). In contrast, PF manifests with small, superficial blisters that rapidly develop into crusted erosions, commonly in seborrheic regions like the face, and scalp [22].

Diagnosis relies on histopathological and DIF analysis. A lesional biopsy for histopathology involves a 3- to 5-mm punch biopsy of a recent vesicle (< 24 h) or a peripheral portion. For DIF, perilesional skin or mucosa biopsies are taken and transported in liquid nitrogen, saline (< 36 h), or Michel’s fixative. Histopathologically, PV shows intraepidermal suprabasal acantholysis with sparse inflammation (Fig. 3b), whereas PF presents acantholysis at the granular layer with more prominent inflammation [96]. DIF typically reveals IgG and/or C3 deposits forming a net-like pattern [97] (Fig. 3c). Although antibody deposition in PF is generally more superficial than in PV, distinguishing PV from PF solely based on DIF can be challenging [98].

### Traditional therapy

Advances in treatment, particularly Corticosteroids and immunosuppressants, have significantly reduced the mortality rate of PV from 75% in the 1950 s to less than 5% today [99]. These improvements underscore the importance of understanding its pathogenesis to optimize therapeutic strategies. Corticosteroids remain the cornerstone of pemphigus treatment and are typically the first-line therapy for both PV and PF, irrespective of disease severity [100]. For mild pemphigus (the pemphigus disease area index (PDAI) score ≤ 15) [101], most guidelines recommend an initial corticosteroid dose of 0.5–1.0 mg/kg/day (prednisolone equivalent). In moderate-to-severe cases (PDAI score > 15), higher doses of 1.0–1.5 mg/kg/day are often prescribed, frequently in combination with adjuvant therapies. If disease control is inadequate, the corticosteroid dose should be increased. Once remission is achieved—defined as ≥ 80% healing of lesions with no new lesions for at least two weeks—the corticosteroid dose should be tapered gradually [102]. Tapering typically begins with a more rapid reduction, slowing over time to minimize relapse risk, with the goal of reducing the dose to < 10 mg/day. In cases of disease flare, characterized by the development of three or more new lesions within a month or the worsening of previously healed lesions, the corticosteroid dose should be increased. However, the exact dosage adjustments are not standardized across all guidelines and consensus [102].

Immunosuppressive agents are commonly used as adjuvants in pemphigus treatment. Mycophenolate mofetil (MMF) and azathioprine (AZA) are the most frequently recommended options, with MMF often preferred as a first-line adjuvant. Other alternatives include mycophenolic acid (MPA), methotrexate (MTX), cyclophosphamide (CTX), cyclosporine A (CsA), and dapsone (the latter primarily for PF) [100]. Dosages of immunosuppressive agents may vary slightly among guidelines. Adverse drug reactions should be monitored, and drug-related genetic testing (e.g., for AZA) is recommended where applicable [103]. For severe or refractory cases, additional treatments such as intravenous immunoglobulin (IVIG), plasma exchange, and immunoadsorption may be employed.

### Targeted therapy

Although traditional therapies have demonstrated efficacy, their associated adverse effects and risks significantly burden patients [104]. The emergence of targeted therapies offers a promising alternative, focusing on specific immune mechanisms to mitigate autoimmune responses while minimizing systemic effects. Rituximab (RTX) a chimeric anti-CD20 monoclonal antibody, targets the CD20 antigen on pre-B and mature B lymphocytes [105]. By inducing complement-dependent cytotoxicity (CDC) and antibody-dependent cell-mediated cytotoxicity (ADCC), RTX depletes B cells, which are central to the autoimmune response in pemphigus [106]. Initially used for refractory pemphigus in 2002 [107], RTX’s efficacy was confirmed by a prospective study in 2007 [108]. To date, over 500 cases of RTX-treated pemphigus have been reported [105]. When combined with corticosteroids and immunosuppressants, RTX facilitates safe corticosteroid tapering [109, 110]. However, relapses occur in approximately 40% of patients, with a mean remission duration of 15–17 months [109]. A landmark randomized controlled trial in 2017 demonstrated a significantly higher complete remission rate (89%) with RTX and short-term corticosteroids compared to corticosteroid monotherapy (34%) [111]. Based on these results, RTX was approved as first-line therapy for moderate-to-severe pemphigus in the US (2018) and Europe (2019). Common dosing regimens include four infusions of 375 mg/m^2^ weekly or two infusions of 1000 mg two weeks apart [105]. Recent studies suggest low-dose RTX (e.g., a single 500 mg infusion) may be effective with fewer side effects [112]. Ofatumumab (OFA), a fully human anti-CD20 monoclonal antibody, induces stronger CDC and ADCC than RTX [113, 114], making it a viable option for patients resistant to RTX. The first successful OFA treatment in pemphigus was reported in 2018 [115]. Subsequent studies, including a 36-week follow-up study, suggest OFA as a promising alternative [116, 117]. Veltuzumab, another anti-CD20 antibody, has also been used, though less extensively studied than OFA [118].

Other emerging treatments for pemphigus are also worthy of attention and expectation. Bruton's tyrosine kinase (BTK) plays a critical role in B-cell activation, making it a target for therapies that prevent B-cell activation without depleting them [119]. Rillzabrutinib, a potent BTK inhibitor, showed promise in a Phase II trial, with six patients achieving complete response [120]. However, the subsequent Phase III PEGASUS trial failed to meet its primary endpoint [121]. Despite this, the Phase II results encourage further investigation. The B-cell activating factor (BAFF) is crucial for B-cell survival and differentiation, making it a potential target for pemphigus therapy [122]. BAFF inhibitor (NCT01930175) shows potential for pemphigus therapy, with early Phase II trial results indicating efficacy [123]. Efgartigimod, a FcRn-blocking monoclonal antibody reduces autoantibody levels [124]. A Phase III trial (NCT04598451/NCT04598477) did not meet its primary endpoint, though promising results in earlier studies warrant further exploration [125]. Tofacitinib is a Janus kinase (JAK) inhibitor that holds promise in targeted therapy for pemphigus due to its ability to modulate immune signaling pathways involved in autoantibody production, Preliminary studies suggest it is effective in mild-to-moderate cases [123]. Chimeric Autoantibody Receptor T Cells (CAART): Dsg3-CAART cells, designed to target pathogenic memory B cells expressing anti-Dsg3 B cell receptors [126, 127], have shown positive results in preclinical and early-phase clinical studies (NCT04422912), with ongoing research to optimize dosing and assess long-term outcomes. Targeted therapies, while still evolving, hold significant promise for reducing treatment burdens and improving outcomes in pemphigus patients.

### Multidisciplinary care

Pemphigus is a chronic, relapsing disease that requires ongoing monitoring of clinical symptoms and the side effects of immunosuppressive therapy. Given its complexity, multidisciplinary care (MDC) is critical for ensuring comprehensive patient management. This approach involves collaboration among specialists to monitor disease activity, treatment side effects, and overall patient well-being. Infections, particularly pneumonia and sepsis, remain the leading causes of mortality in pemphigus patients [128]. Vigilant surveillance and infection prevention are essential, especially during pandemics such as COVID-19. While our study reported that immunosuppressants, particularly low-to-medium doses of glucocorticoids, did not significantly increase COVID-19 risk in pemphigus patients [129], careful medication management and infection control are still paramount.

Clinical monitoring is a cornerstone of pemphigus management and should involve regular follow-up visits. During the initial treatment phase, follow-ups every 2–4 weeks are recommended until disease control is achieved. Once corticosteroid doses are tapered, visits can be spaced out to every 4–8 weeks and eventually to every 8–16 weeks or even longer for patients in remission. Dermatologists play a central role in assessing disease activity using tools such as the PDAI or autoimmune bullous skin disorder intensity score (ABSIS). They also monitor serum levels of anti-Dsg1 and anti-Dsg3 autoantibodies via ELISA to evaluate therapeutic response [98]. Except for dermatologists, internal medicine specialists monitor side effects of immunosuppressive therapies, including diabetes, hypertension, cardiac insufficiency, infections, and hematological abnormalities. Dermatologists and oral physicians assess skin and mucosal lesions, while ophthalmologists evaluate ocular involvement. Pharmacists ensure adherence to prescribed therapies and monitor for drug interactions and adverse effects. Psychologists address the psychological challenges of living with chronic disease, including mental health concerns and pain management. The effectiveness of multidisciplinary care in pemphigus has been demonstrated in various case reports and studies [130, 131]. This model, involving dermatologists, oral physicians, ophthalmologists, pain management specialists, psychologists, surgeons, and others, has proven beneficial for both patients and healthcare providers, improving outcomes and optimizing care delivery [132, 133].

## Paraneoplastic pemphigus

PNP, first described in 1954 as a neoplasm-associated pemphigus-like disorder [134], has undergone significant nosological refinement. Initially classified as a pemphigus subtype due to overlapping blistering lesions, the discovery of its multiorgan involvement—including fatal obliterative bronchiolitis and lichenoid mucocutaneous eruptions—led to its redefinition. In 2001, scholars proposed renaming PNP as PAMS to reflect its heterogeneous autoimmune manifestations beyond epithelial adhesion defects [135]. Although PNP shares superficial similarities with classical pemphigus (e.g., autoantibody-mediated epithelial detachment), it is now recognized as a distinct entity within the AIBD spectrum due to some key features: i.e., (1) pathogenic complexity: unlike pemphigus vulgaris or foliaceus, which primarily target desmosomal proteins (e.g., Dsg1/Dsg3), PNP involves autoantibodies against both desmosomal (e.g., desmoplakin, envoplakin, and periplakin) and BMZ components (e.g., BP230 and BP180); (2) immune mechanism heterogeneity: PNP pathogenesis involves both humoral and cell-mediated immunity, with cytotoxic T-cell infiltration contributing to epithelial destruction; (3) strong neoplastic association: Over 80% of PNP cases are linked to lymphoproliferative or hematologic malignancies (e.g., Castleman disease, chronic lymphocytic leukemia), necessitating oncologic evaluation in diagnosis and management [136]. The following subsections detail PNP’s pathogenesis, clinical characteristics, therapeutic strategies, and the critical role of multidisciplinary care in addressing its systemic manifestations.

### Epidemiology, genetic and environmental factors

PNP is exceptionally rare, with an estimated annual incidence of fewer than one case per million population [137]. Genetic predisposition plays a role, with specific HLA alleles identified as risk factors [138]. For example, the HLA-DRB1*03 allele has been linked to PNP in French patients, while the *HLA-Cw14* allele has shown an association in Chinese populations, where HLA-DRB1*03 is not detected [139]. The tumor-induced pathogenesis of PNP has been widely accepted, whereas potential environmental determinants remain poorly characterized due to limited cases.

### Pathogenesis of paraneoplastic pemphigus

The pathogenesis of PNP is primarily driven by autoantibodies targeting tumor antigens that cross-react with epithelial antigens, disrupting both desmosomal and hemidesmosomal structures. Key target antigens in PNP include plakin family proteins (plectin, desmoplakins I and II, envoplakin, periplakin, and BP230), as well as desmogleins 1 and 3 [140, 141]. Alpha-2-macroglobulin-like protein 1 (A2ML1) has also been identified as a target antigen [142]. The autoantibodies implicated in PNP are often produced by B cell clones associated with underlying neoplasms, such as Castleman disease, thymoma, and follicular dendritic cell sarcoma [143–145]. Immune cells are central to PNP pathogenesis, with evidence suggesting that cell-mediated immunity may precede and promote humoral responses [146, 147]. Skin lesions from PNP patients show significant immune cell infiltration [135], and pro-inflammatory cytokines, including interferon-gamma and tumor necrosis factor-alpha (TNF-α), are implicated in epithelial damage [148].

Plakins, especially envoplakin and periplakin, are critical autoantigens in PNP [149, 150]. These proteins share a characteristic structure that includes an amino-terminal globular domain, a central coiled-coil rod domain, and a carboxy-terminal tail containing repeating amino acid domains [151]. The homologous region in the carboxy terminus of plakins is a key antigenic site. Additional antigenic domains [143, 152, 153], such as EVPL-N1 (aa1–141) and EVPL-L3 (aa1684–1784) in envoplakin, and PPL-N1 (aa1–102) and PPL-N3 (aa201–324) in periplakin, have demonstrated high sensitivity and specificity for detecting PNP autoantibodies [154]. These advances have promoted the development of serological diagnostic techniques, diagnostic techniques such as immunoprecipitation and ELISA using full-length recombinant proteins have proven effective in identifying anti-periplakin and anti-envoplakin antibodies, enhancing diagnostic accuracy [155].

### Characteristics of paraneoplastic pemphigus

Although serum from PNP patients recognizes Dsgs, the epitope distribution in PNP is broader than in classic pemphigus. In PNP, the epitopes within the extracellular domain of Dsg3 are more diffusely distributed, contrasting with the more localized recognition pattern in pemphigus [90]. Additionally, PNP is characterized by epitope spreading, which can result in the detection of nonpathogenic antibodies, such as those targeting Dsg1 and Dsg3, which do not contribute to disease pathogenesis. Diagnosis of PNP relies on a combination of clinical manifestations and laboratory findings. Unlike pemphigus, PNP often presents chronic erosive mucositis, polymorphic skin lesions, lichenoid dermatitis, and erythema multiforme-like lesions. The presence of these features, particularly mucositis, can aid in differentiation. PNP is also strongly associated with neoplasms, most commonly lymphoproliferative or hematologic malignancies. Histopathologically, PNP is characterized by lichenoid interface dermatitis, acantholysis, or keratinocyte necrosis. DIF typically reveals deposits of IgG and C3 in an intercellular pattern within the epidermis or a linear pattern along the BMZ. Lichenoid inflammation may expose cell surface and BMZ antigens, triggering the production of autoantibodies that are hallmark features of PNP [146, 147]. Recent studies have also shown that lichenoid/interface dermatitis is associated with prognosis in PNP patients [156]. Serological testing is crucial for diagnosis. Indirect immunofluorescence (IIF) using rat bladder tissue can detect epithelial cell membrane and/or cytoplasmic staining. ELISA, immunoblotting, or immunoprecipitation techniques are used to identify antibodies targeting envoplakin, periplakin, desmoplakin, or A2ML1 proteins. For patients diagnosed with PNP without an identified neoplasm, regular monitoring is recommended to detect occult malignancies [137].

**Fig. 4:**
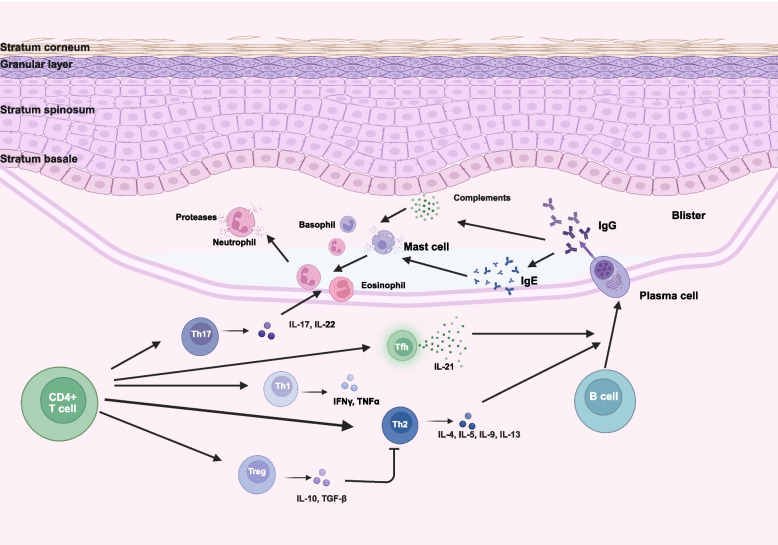
Pathogenesis of bullous pemphigoid. This picture shows the pathogenesis of bullous pemphigoid (BP). BP is characterized by the presence of IgG autoantibodies and complement component C3, which target the dermoepidermal basement membrane—the adhesion structure of the epidermis. Immune complex formation initiates complement activation, recruiting mast cells, neutrophils, and eosinophils, and triggering the release of proteases and inflammatory mediators, leading to dermal–epidermal separation. Th17 cells infiltrate skin tissues, where their production of IL-17 activates neutrophils, amplifies the inflammatory response, and causes tissue damage. Th2 cells and IL-4 promote B cell proliferation, antibody production, and immunoglobulin class switching. Pathogenic Th17 cells exacerbate the inflammatory immune response, while dysregulated Treg cells lead to spontaneous activation of autoreactive CD4 + T cells and promote autoantibody production. Increased Tfh cell activation aids B cells in producing autoantibodies in BP. Created in https://BioRender.com

### Traditional therapy

Management of PNP typically involves addressing the underlying malignancy. Early detection and radical resection of tumors, such as Castleman disease or thymoma, can significantly improve outcomes [157, 158]. Pharmacologically, corticosteroids remain the cornerstone of treatment, administered at doses ranging from 0.5–1.5 mg/kg per day. While effective for cutaneous lesions, corticosteroids may be less efficacious for mucositis or bronchiolitis obliterans, both of which frequently complicate PNP. Adjuvant therapies, including AZA, MTX, and CsA, have been used to enhance therapeutic outcomes, particularly for patients requiring prolonged immunosuppression [159, 160]. Thalidomide has shown promise in certain cases [161], while high-dose IVIG provides temporary relief for refractory skin and mucosal lesions [159].

### Target therapy

For PNP associated with B-cell malignancies, therapies targeting B cells, such as RTX, are commonly employed. RTX, an anti-CD20 monoclonal antibody, is particularly effective in patients with hematologic malignancies, as it reduces autoantibody production [162, 163]. In refractory cases, low-dose anti-CD52 therapy with alemtuzumab has shown promising results [164–166]. Monoclonal antibodies targeting T cells, such as daclizumab (anti-CD25), have demonstrated efficacy in treating bronchiolitis obliterans associated with PNP [167], especially in cases linked to chronic lymphocytic leukemia. bronchiolitis obliterans remains a major, life-threatening complication of PNP, often unresponsive to aggressive immunosuppressive therapy. For mild BO, treatments such as tocilizumab (anti-IL6), alemtuzumab, RTX, or ibrutinib may be beneficial. However, severe cases often require lung transplantation [168].

### Multidisciplinary care

PNP is a complex, systemic condition that affects multiple organs, including the lungs, thyroid, kidneys, smooth muscle, and gastrointestinal tract [169]. Lung involvement, such as bronchiolitis obliterans, is particularly severe and often life-threatening. Additionally, gastrointestinal complications, such as erosions, may occur. Given its systemic nature, PNP requires a comprehensive, multidisciplinary approach. Dermatologists, hematologists/oncologists, pulmonologists, gastroenterologists, and infectious disease specialists must collaborate to optimize treatment outcomes with minimizing adverse effects. The immunosuppressive therapies used to manage PNP heighten the risk of opportunistic infections, necessitating close monitoring. The value of multidisciplinary care has been highlighted in case reports where aggressive PNP was successfully managed through coordinated efforts among specialists [170]. This underscores the importance of integrating diverse expertise into routine clinical practice to improve patient outcomes. Clinicians should advocate for team-based care models and institutional support to address the challenges of managing this complex disease effectively.

## Pemphigoid disease

Pemphigoid diseases comprise a group of subepidermal autoimmune blistering disorders characterized by autoantibodies targeting key structural components of the BMZ, such as plectin, BP230, collagen XVII/BP180, α6β4 integrin, laminin 332, and p200 (laminin γ1 or laminin β4) [23]. Major subtypes include bullous pemphigoid (BP), mucous membrane pemphigoid (MMP), epidermolysis bullosa acquisita (EBA), anti-p200 pemphigoid, and linear IgA bullous dermatosis (LABD) [96]. This section focuses on the epidemiology, genetic and environmental factors, pathogenesis, and therapy of BP, MMP, EBA, and anti-p200 pemphigoid, while LABD will be discussed in IgA-related AIBDs section due to the unique IgA-mediated pathological mechanism.

### Epidemiology, genetic and environmental factors

Bullous pemphigoid (BP) is the most prevalent subtype of pemphigoid diseases, with a mean age of onset between 66 and 83 years. Its incidence increases significantly with age, with an estimated annual rate of 2.4–21.7 new cases per million population worldwide [171]. MMP primarily affects mucous membranes and is characterized by chronicity and scarring tendencies [2]. Its annual incidence is estimated at 1–2 new cases per million in Germany and France, predominantly affecting individuals aged 60–80 years at diagnosis [172]. EBA is a rare subtype, with incidence rates ranging from 0.08 to 0.5 cases per million population. Similarly, anti-p200 pemphigoid, first described in 1996, remains rare, and epidemiological data are limited [173, 174].

Genetic predisposition plays a critical role in pemphigoid diseases. HLA class II alleles, such as DQB1*0301 in Caucasians, have been associated with BP [175]. In the Japanese population, associations with DRB1*04, DRB1*1101, and DQB1*0302 alleles have been identified [176, 177]. EBA has shown links to *HLA-DR2* in African Americans, HLA-DRB1*15:03 in African-descended populations in France, and HLA-DRB1*13 in Koreans [178–180]. Environmental triggers include medications (e.g., antibiotics, beta-blockers, non-steroidal anti-inflammatory drugs (NSAIDs), diuretics, dipeptidyl peptidse-4 (DPP-4 inhibitors), and immune checkpoint inhibitors targeting programmed cell death protein 1 (PD-1) and its ligand (PD-L1)), infections, vaccines, trauma, surgical interventions, burns, ultraviolet (UV) exposure, radiotherapy, and photodynamic therapy. These factors may either initiate or exacerbate the disease [181]. For some subtypes, such as anti-p200 pemphigoid, data are lacking due to their low incidence.

### Pathogenesis of pemphigoid

Pemphigoid diseases are autoimmune disorders characterized by immune responses targeting BMZ proteins. Triggers such as medications or infections disrupt immune tolerance, leading to autoreactive T and B cell activation and subsequent IgG or IgA autoantibody production [182, 183]. These autoantibodies bind to BMZ antigens, activating the complement cascade and recruiting granulocytes, which release proteolytic enzymes, causing dermo-epidermal separation and blister formation [184]. In the complement-dependent pathway, IgG autoantibodies bind BMZ antigens, activating complement components (primarily C3) via Fc receptors. This triggers recruitment of neutrophils and mast cells, which degranulate to recruit eosinophils and neutrophils, releasing enzymes that degrade BP180 and extracellular matrix proteins [185–188]. Concurrently, inflammatory cytokines like IL-4, IL-13, and IL-31 promote pruritus and inflammation. In some cases, autoantibodies independently stimulate keratinocytes to release inflammatory cytokines and reduce adhesion by inducing BP180 internalization [189]. T cell subsets play pivotal roles. Th2 cytokines (IL-4, IL-13, and IL-31) promote B cell activation, eosinophil recruitment, and pruritus [190, 191]. The IL-17/IL-23 axis has been implicated in BP pathogenesis [183], where IL-17 enhances protease activity, contributing to tissue destruction [192–194]. T follicular helper (Tfh) cells also stimulate autoantibody production by promoting B cell differentiation [195–197]. (Fig. 4).

The pathogenesis of anti-p200 pemphigoid remains unclear but may involve autoantibodies disrupting laminin–integrin interactions in the BMZ, potentially through inflammatory pathways overlapping with psoriasis [198–200]. In MMP, IgG autoantibodies target laminin 332 and integrin α6β4, causing chronic mucosal inflammation and scarring. Autoreactive T cells facilitate B cell differentiation and autoantibody production [201]. EBA is driven by a loss of tolerance to type VII collagen. Autoreactive T and B cells collaborate to produce autoantibodies, which mediate tissue injury through Fc-dependent mechanisms. Neutrophil-driven inflammation, along with the release of reactive oxygen species and proteases, exacerbates tissue damage and blister formation [202–204]. The heterogeneity of pemphigoid diseases arises not only from distinct autoantibody profiles but also from the specific antigens and epitopes targeted, which critically determine clinical phenotypes, disease severity, and therapeutic responsiveness.

### Antigens and epitopes

Pemphigoid diseases are autoimmune blistering disorders marked by autoantibodies targeting components of the BMZ. This section reviews key antigens implicated in the pathogenesis of pemphigoid diseases, their major epitopes, and their roles in diagnosis and disease progression. Autoantibodies against BP180 and BP230 are hallmarks of BP. BP180, also known as BP antigen 2 or type XVII collagen, is a transmembrane protein with a globular cytoplasmic domain and an extracellular region containing 15 collagenous domains interspersed with non-collagenous (NC) domains [205–208]. The NC16 A domain (aa 490–562), located at the start of the extracellular region [209, 210], is the principal epitope in BP. IgG, IgA, and IgE autoantibodies targeting NC16 A are commonly found in BP patient sera [211, 212]. However, IgE autoantibodies to NC16 A have shown no correlation with disease activity [213]. Additional antigenic sites exist within both intracellular and extracellular regions of BP180. Studies using recombinant fragments have identified new epitopes beyond NC16 A, including those at the C-terminus, particularly in DPP-4i-induced BP [214]. Between 7.8% and 47% of BP sera recognize recombinant BP180 ectodomain fragments excluding NC16 A [215–217], while 28%–82% of sera react with intracellular epitopes [215, 218, 219]. The pathogenic significance of these epitopes requires further investigation. BP230, or BP antigen 1, is an intracellular hemidesmosomal protein belonging to the plakin family [220]. It links keratin filaments to BP180 and the β4 integrin subunit. Early research identified BP230 as a major BP antigen through immunoprecipitation and immunoblotting [221–223]. Subsequent studies have mapped several epitopes [224–229], including FP3 (aa 1003–1193), FP7 (aa 1623–1812), and rBP55 (aa 1722–2201), with significant variability in epitope recognition across different populations. Antibodies against BP230 are detected in BP and PNP [230–232].

A 200-kDa glycoprotein in the lamina lucida of the BMZ is the antigen in anti-p200 pemphigoid [173, 233]. Initial studies identified the protein using patient sera [173, 233], and later research pinpointed the C-terminal region (aa 1364–1609) as immunodominant [198, 234]. However, murine models suggest the pathogenicity may reside in the N-terminal domain [235, 236]. N-linked glycosylation of laminin γ1 also appears to influence antibody recognition [237]. Laminin 332 (laminin 5) is an extracellular matrix protein composed of disulfide-linked α3, β3, and γ2 subunits [238–242]. It binds integrin α6β4 and supports BMZ adhesion. Laminin 332 is a target antigen in MMP [243, 244], with most patient sera recognizing the α3 subunit [245]. Immunogenic domains within the α3 G regions (aa 970–1713) have been identified [246], though not all studies have confirmed these findings [247]. Limited data exist on β3 and γ2 subunit epitopes [244, 248]. Some ELISA kits were developed using purified laminin 332 from squamous carcinoma cells (SCC) [241, 249]. Integrins are cell surface receptors involved in cell adhesion, signal transduction, and hemidesmosome assembly. The α6β4 integrin, a BMZ heterodimer, is a target antigen in MMP [20, 250, 251]. Identified epitopes include aa 292–305 of the α6 subunit [252] and specific fragments of the β4 intracellular (IC) domain [252], such as IC3.4 (aa 1489–1572) for ocular cicatricial pemphigoid (OCP) and IC3.6 (aa 1573–1822) for MMP [253]. Type VII collagen is a BMZ structural protein crucial for dermo-epidermal adhesion. It consists of three alpha-helical chains flanked by NC1 and NC2 domains. Autoantibodies targeting the NC1 and NC2 regions are characteristic of EBA [254–257]. The FP4 fragment of NC1 (aa 814–1439) is recognized by most sera in EBA patients [258–261]. Diagnostic performance of type VII collagen-specific serological assays such as NC1/NC2 ELISA and immunoblotting demonstrating high sensitivity [262–264].

### Characteristics of pemphigoid

The clinical presentation of pemphigoid subtypes varies considerably. BP, the most common subtype, is characterized by tense blisters that often heal without scarring or milia formation. These blisters typically develop on an erythematous or urticarial background and predominantly affect the distal extremities, trunk, and intertriginous areas (Fig. 3d).

In contrast, MMP is more severe due to its extensive mucosal involvement, which can affect the oral cavity, eyes, nasopharynx, larynx, trachea, and anus. Oral lesions frequently present gradual onset, spontaneous recurrence, and ulceration, commonly affecting the gingiva (80%), buccal mucosa (58%), palate (26%), alveolar ridge (16%), tongue (15%), and lower lip (7%). Ocular involvement in MMP typically manifests as chronic conjunctival inflammation, which can progress to vision loss if untreated, accompanied by symptoms such as redness, tearing, burning, and a foreign body sensation.

Nasal symptoms in MMP often include epistaxis, rhinorrhea, crusting, and nasal congestion. Physical examination may reveal atrophic rhinitis, erosive and crusting lesions, or adhesions. Pharyngeal involvement is marked by symptoms such as sore throat, dysphagia or odynophagia, impaired food intake, and cough. Nasopharyngeal and laryngeal involvement frequently coexist, presenting with erythema, erosions, ulcers, blistering lesions, and pharyngeal scarring. Laryngeal involvement can lead to dyspnea and dysphonia, though some patients may remain asymptomatic. Esophageal involvement often develops years after disease onset, manifesting as dysphagia and potentially progressing to strictures. Skin involvement in MMP is less common, occurring in approximately one-third of cases, and is typically limited to crusts, erosion, and scarring on the head and neck. Without appropriate treatment, MMP can result in severe complications, including blindness, difficulty breathing or swallowing, and, in extreme cases, death.

EBA is characterized by skin fragility, vesicles, tense blisters, and erosions on non-inflamed skin, often healing with scarring and milia formation. The condition can affect any area of the skin and mucous membranes, with trauma-prone regions such as the dorsal hands, elbows, and knees being especially vulnerable. Cicatricial alopecia and onychodystrophy are additional manifestations. Anti-p200 pemphigoid commonly presents with tense blisters and urticarial eruptions, resembling either BP or the inflammatory form of EBA.

Histopathological examination of lesional skin in pemphigoid patients typically reveals subepidermal bullae with inflammatory cell infiltration (Fig. 3e). In BP, this infiltrate primarily consists of eosinophils or neutrophils, often accompanied by dermal infiltrates or eosinophil margination along the dermo-epidermal junction. In EBA and anti-p200 pemphigoid, the infiltrate is predominantly neutrophilic, whereas MMP exhibits a mixed inflammatory infiltrate, including monocytes, histiocytes, plasma cells, eosinophils, and neutrophils. DIF typically demonstrates linear deposition of IgG and/or C3 at the dermo-epidermal junction, with occasional detection of IgA or IgE in a similar pattern (Fig. 3f). EBA may occur in conjunction with systemic lupus erythematosus (SLE), giving rise to the term"bullous SLE,"which shares similar clinical features and DIF results with AIBDs. Distinguishing bullous SLE from other AIBDs requires additional SLE-specific examination.

Serological testing can identify anti-BP180 and anti-BP230 IgG autoantibodies in BP patients. IIF demonstrates serum IgG autoantibodies binding to the dermal side of salt-split skin in EBA and anti-p200 pemphigoid, while binding to the epidermal side in BP and anti-BP180-type MMP. Anti-p200 IgG autoantibodies can also be detected using immunoblotting or ELISA. Similarly, autoantibodies against laminin 332 or integrin α6β4 are detectable in patients’ serum. Additionally, anti-type VII collagen antibodies can be identified using ELISA or immunoblotting. However, commercial ELISA kits are currently limited to detecting BP180, BP230, and type VII collagen autoantibodies.

### Traditional therapy

Corticosteroids are the cornerstone of treatment for pemphigoid. For patients with mild to moderate BP, defined by a BP disease activity index (BPDAI) score of < 20 and 20–57, respectively, a daily dose of 0.5 mg/kg of prednisone is recommended. In severe cases (BPDAI score ≥ 57), the same starting dose is advised, with an increase to 0.75 mg/kg/day if disease control is not achieved within three weeks. Super-potent topical corticosteroids are also an effective treatment option [265]. In EBA or anti-p200 pemphigoid, initial corticosteroid doses range from 0.5 to 2.0 mg/kg/day [266, 267]. For patients resistant to corticosteroids or those experiencing relapses, immunosuppressive agents such as AZA, MTX, or MPA are effective alternatives [268–271]. Treatment for MMP differs from other pemphigoid diseases [272]. Mild MMP, characterized by isolated oral or ocular involvement, is typically managed with dapsone, tetracyclines, sulfasalazine, or prednisolone at 0.3–0.5 mg/kg/day. In more severe cases without significant ocular, tracheal, laryngeal, or esophageal involvement, prednisolone 0.5–1.0 mg/kg/day combined with MTX or AZA is recommended.

### Target therapy

Targeted therapies, including biologics and JAK inhibitor (JAKi), are emerging as effective options for refractory pemphigoid. AIBDs often involve autoreactive B cells, making them primary therapeutic targets. Anti-CD20 monoclonal antibodies, such as RTX, have been used in pemphigoid [273], though their effectiveness in BP appears lower than in pemphigus regarding disease control, remission rates, and relapse prevention. Anti-BAFF monoclonal antibodies, which modulate B-cell survival and function, are also being explored as potential treatments [274].

Inflammation plays a significant role in BP pathogenesis, and recent studies have demonstrated the efficacy and safety of dupilumab [275, 276], an interleukin-4 receptor alpha antagonist. For BP patients with urticarial lesions and elevated serum IgE, Omalizumab, an anti-IgE monoclonal antibody, has shown promise in preliminary studies [277]. JAKis have also emerged as viable options for BP, with evidence supporting their efficacy in reducing disease severity [278, 279]. In MMP, JAKi and anti-TNF-α therapies are under investigation, with ongoing studies evaluating their potential in disease management [280, 281]. Additional therapeutic targets in BP include the IgG1 Fc fragment, eosinophils, neutrophils, Th17 cytokines, the TWEAK/Fn14 pathway, and proteases. Efgartigimod, a novel IgG1 Fc fragment, has shown potential in clearing autoantibodies [282], and the phase 2/3 BALLAD trial is currently evaluating its efficacy in moderate-to-severe BP. Future therapies, such as CAART and microRNA-targeting treatments, aim to provide precise disease regulation [283].

### Multidisciplinary care

Pemphigoid is a chronic, multisystem disease requiring careful monitoring and interdisciplinary management to address clinical symptoms and the side effects of immunosuppressive therapy. Regular follow-up visits are essential for assessing disease activity, adjusting therapy, and determining the appropriate time for treatment discontinuation. Disease monitoring should include BPDAI or ABSIS scoring, along with measurements of anti-BP180 and anti-BP230 antibody levels. Management of adverse drug reactions (ADRs) and pemphigoid-related complications requires collaboration among specialists, including dermatologists, ophthalmologists, ear, nose, and throat (ENT) specialists, gastroenterologists, gynecologists, urologists, maxillofacial surgeons, and dentists. Dermatologists manage cutaneous lesions and overall disease progression. Ophthalmologists monitor and treat ocular involvement, preventing complications such as conjunctivitis, corneal scarring, and blindness. ENT specialists address lesions in the nasal and pharyngeal regions. Gastroenterologists manage esophageal or gastrointestinal involvement, including dysphagia and stricture formation. Gynecologists and urologists focus on anogenital lesions to prevent scarring and adhesions. Maxillofacial surgeons treat oral or facial lesions, ensuring proper healing and function. Dentists maintain oral hygiene and manage mucosal ulcerations [272]. Studies highlight the benefits of a collaborative, multidisciplinary approach in improving patient outcomes and physician satisfaction, particularly in managing the diverse manifestations of MMP [130].

## IgA-related autoimmune blistering diseases

IgA-related AIBD, including IgA pemphigus, linear IgA bullous dermatosis (LABD), and dermatitis herpetiformis (DH), are characterized by IgA autoantibodies targeting adhesion components, leading to blister formation. IgA pemphigus is a rare subtype of pemphigus associated with IgA autoantibodies directed against keratinocyte surface components critical for cell adhesion. IgA pemphigus includes two major histopathological and immunological subtypes: intraepidermal neutrophilic IgA dermatosis (IEN)-type and subcorneal pustular dermatosis (SPD)-type [171, 284]. LABD is an IgA-mediated pemphigoid affecting both children and adults. DH), also known as Duhring disease, is another IgA-related AIBD that is caused by gluten sensitivity. This section discusses pathogenesis, clinical characteristics, and treatment of IgA-related AIBDs.

### Epidemiology, genetic and environmental factors

IgA pemphigus can occur across all age groups, from infancy to advanced age, but is most observed in adults, typically between the fourth and sixth decades of life. Due to its rarity, epidemiological data on IgA pemphigus are currently unavailable. LABD is notably the most common pemphigoid in children, with a bimodal age distribution. The first peak occurs below the age of 5, and the second peak is seen in individuals over the age of 60. Epidemiologically, the incidence of LABD is estimated to range between 0.2 and 1.0 cases per million people per year, with variations across different geographical regions [171]. The incidence of DH ranges from 0.4 to 3.5 cases per 100,000 people per year, while its prevalence is reported to range from 11.2 to 75.3 per 100,000 [285]. DH is triggered by gluten sensitivity and genetic predisposition also plays a significant role, as first-degree relatives of DH patients exhibit an almost 15-fold increased risk compared to the general population [286]. Specifically, the HLA-DQB1*0201 and HLA-DQA1*0501 alleles have been identified as risk factors, as they are involved in the processing of the gluten antigen, gliadin [287]. The genetic and environmental factors underlying IgA pemphigus and LABD remain to be elucidated, and further research is needed to determine their contributions to disease pathogenesis.

### Pathogenesis of IgA-related autoimmune blistering diseases

The pathogenesis of IgA pemphigus, LABD, and DH involves IgA autoantibodies, although the targets and mechanisms differ. In SPD-type IgA pemphigus, IgA autoantibodies primarily target desmocollin 1 (Dsc1), while the antigen in IEN remains unclear [288, 289]. In some cases, IgA antibodies recognize the same epitopes as IgG antibodies [290], whereas, in others, they bind distinct domains, indicating a unique immune response [291]. Autoantibodies bind Fc receptors (CD89) on monocytes and granulocytes, leading to neutrophil infiltration and keratinocyte junction cleavage, resulting in blister formation [292]. Epitope spreading exacerbates the disease by exposing new antigens during inflammation [293]. IgA pemphigus is also associated with monoclonal IgA gammopathy and multiple myeloma, though the role of malignancies remains uncertain [294]. In contrast, LABD is caused by IgA antibodies targeting the BMZ. Specifically, BP180 cleavage generates LAD-1 (120 kDa) and LABD97 (97 kDa) fragments, which serve as LABD antigens. These fragments are also implicated in BP and MMP, suggesting shared pathogenic pathways [201, 295, 296].

The pathogenesis of DH is complex and involves interactions among genetics, immunology, and environmental factors. DH is triggered by gluten sensitivity, where gliadin is presented to CD4^+^ T-cells, resulting in inflammation and damage to mucosal epithelial cells. The modified glutamine residues of gliadin also cross-link covalently to tissue transglutaminase (TG2/tTG), forming a complex that is presented to gliadin-specific helper T-cells. These T-cells then stimulate B-cells to produce circulating IgA antibodies directed against TG2, which is also the antigen in celiac disease. Some researchers suggest that DH may be an extraintestinal manifestation of celiac disease [297]. Through epitope spreading, circulating IgA autoantibodies also target epidermal transglutaminase (TG3/eTG) found in the skin. This forms a complex with TG3 produced by keratinocytes, leading to an inflammatory response [298].

### Characteristics of IgA-related autoimmune blistering diseases

Patients with IgA pemphigus often present with vesicles or blisters that evolve into pustules on the trunk, extremities, scalp, retroauricular regions, or intertriginous areas [292]. Histopathology reveals mild acantholysis and neutrophilic infiltration. In the SPD subtype, pustules are localized subcorneally in the upper epidermis, while in the IEN subtype, pustules are suprabasilar and may involve the lower or entire epidermis [289]. DIF reveals IgA deposition in an intercellular pattern, with localization varying by subtype [288, 299]. In LABD, tense bullae often appear in a “cluster of jewels” configuration or as grouped papulovesicles. Mucosal involvement, including oral and ocular erosions, is common. Histopathology shows subepidermal cleavage with dense neutrophilic infiltration. DIF demonstrates linear IgA deposition along the BMZ, aiding in diagnosis. Circulating IgA autoantibodies can also be detected through immunoserological assays, such as immunoblotting [300]. In DH, eroded and crusted papulovesicles or blisters are typically found on the extensor surfaces and buttocks, distributed symmetrically, and are associated with intense itching [301]. Mucosal involvement is rare in DH. Histopathological examination reveals subepidermal separation, with granulocytes (primarily neutrophils and a few eosinophils) forming papillary microabscesses [302]. DIF shows granular or fibrillar IgA deposition along the BMZ [303]. To differentiate DH from LABD, further serologic testing for anti-TG2 or anti-TG3 antibodies is necessary [304].

### Traditional and target therapy

The first-line treatment for IgA pemphigus typically involves corticosteroids, with doses ranging from 0.5 to 1 mg/kg/day, though the disease often demonstrates limited responsiveness to glucocorticoids [305]. In LABD, corticosteroids can be used adjunctively with dapsone for improved disease control [306]. In contrast, corticosteroids are not effective enough in treating DH [307]. Dapsone is the first-line treatment for IgA-related AIBD due to its ability to target neutrophilic infiltration. The typical adult dose ranges from 50 to 150 mg/day [308], while pediatric doses range from 0.5 to 2 mg/kg/day [309]. If dapsone proves ineffective or causes side effects, alternatives include sulfonamides (e.g., sulfapyridine, sulfamethoxypyridazine) and immunosuppressive agents such as colchicine, methotrexate, cyclosporine, or cyclophosphamide. In addition to dapsone, a lifelong gluten-free diet (GFD) is essential for achieving an excellent prognosis in DH [307].

Emerging targeted therapies offer promising alternatives. Adalimumab (anti-TNF-α antibody) and RTX have shown efficacy in refractory cases of IgA-related AIBD [310, 311], while infliximab has been reported to trigger DH [312]. Additional treatments under investigation include erythromycin and combinations of nicotinamide/niacinamide with tetracyclines [313].

## Conclusion and perspectives

This review highlights recent advancements in the pathological mechanisms and clinical management of AIBDs, focusing on the distinct characteristics of various subtypes from the perspectives of immune cells, antigenic targets, and other contributing factors (Tables 1 and 2). It also provides an overview of the progression from conventional immunotherapies to targeted treatments, aiming to offer new insights for clinicians and researchers while fostering further advancements in the diagnosis and treatment of AIBDs, especially in leveraging molecular biomedicine to unravel disease-specific autoantibody signatures, decode microenvironmental crosstalk via multi-omics integration, and engineer antigen-specific biologics that precisely disrupt pathogenic immune circuits, thereby bridging the gap between mechanistic discovery and transformative clinical translation.

**Table 1:** Characteristics and clinical management of autoimmune bullous diseases

	Pemphigus		PNP	Pemphigoid				IgA-related AIBD		
	PV	PF		BP	MMP	EBA	Anti-p200 pemphigoid	IgA pemphigus	LABD	DH
Epidemiology	Pooled incidence rate: 2.83 pmp/y [24]	Incidence rate: < 1pmp/y [22]	Incidence rate: < 1pmp/y [132]	Incidence rate: 2.4–21.7pmp/y [171]	Incidence rate: 1pmp/y [172]	Incidence rate: 0.08–0.5pmp/y [171]	No data	No data	Incidence rate: 0.2-1pmp/y [239]	Incidence rate: 0.4–3.5pmp/y [285]
Genetic factors	HLA-DQB1*03:03, *03:02, *0402, *1401, *1404, and *0503^*99*^	HLA-DRB1*0101, *04^*99*^	HLA-Cw14, HLA-DRB1* 03^*99*^	HLA- DQB1*0301, *0302, HLA-DRB1*04, *1101^*171*^	HLA-DRB1*1503^*171*^	HLA-DR2, HLA-DRB1*15:03, *13^*171*^	No data	No data	No data	HLA-DQB1*0201, DQA1*0501^*287*^
Environmental factors	Medications, infections, stress, diet, immunizations, and sleep [37]	Living environment [40]	No data	Medications, infections, vaccines, and physical factors [181]				No data	Medications, infections, vaccines, and physical factors	Gluten [285]
Histopathology	Intraepidermal suprabasal acantholysis with sparse inflammatory infiltrate	Acantholysis at the granular layer with significant inflammatory infiltrate	Lichenoid interface dermatitis and/or acantholysis and/or keratinocyte necrosis	Subepidermal bullae with eosinophilic infiltration	Subepidermal bullae with mixed inflammatory cell infiltration	Subepidermal bullae with neutrophil infiltration	Subepidermal bullae with neutrophil infiltration	Mild acantholysis and neutrophilic infiltration	Subepidermal cleavage with a dense neutrophilic infiltration	Subepidermal separation with neutrophilic infiltration
Immunopathology	IgG and/or C3 deposits intercellularly		IgG and/or C3 deposits intercellularly or linearly at BMZ	Linear deposition of IgG and/or C3 at the BMZ				IgA and/or C3 deposits intercellularly	Linear deposition of IgA and/or C3 at the BMZ	Granular or fibrillar IgA deposits at the BMZ
Antigen	Dsg1 (aa 86–110, aa 196–220, aa 226–250, aa 326–340, and aa 486–520); Dsg3 EC1	Dsg1 EC1 or EC2	Envoplakin (aa 1–141, aa 1684–1784); Periplakin (aa 1–102, aa 201–324)	BP180 (NC16 A domain, C-terminal domains); BP230 (C-terminal domains)	Laminin α3 (aa 970–1713); integrin α6 (aa 292–305, aa 1489–1702); integrin β4 (aa 1489–1572, aa 1573–1822)	Type VII collagen NC1 or NC2	Laminin γ1, Lamininβ4 (C-terminal domain)	Dsg1, Dsg3, Dsc1	LAD-1; LABD97	Epidermal transglutaminase; tissue transglutaminase
First-line traditional therapy	Mild: CS at 0.5–1.0 mg/kg/day; moderate-to-severe: 1.0 to 1.5 mg/kg/day [98]		Radical resection of tumors, CS at 0.5–1.5 mg/kg/day [137]	CS at 0.5–1.0 mg/kg/day [265]	Mild: CS at a dose of 0.3–0.5 mg/kg/day; moderate: CS at a dose of 0.5–1.0 mg/kg/day with adjuvants; severe: CS at a dose of 1.0–1.5 mg/kg/day with CTX [272];	CS at 0.5 to 2.0 mg/kg/day [267]	CS at 0.5 to 2.0 mg/kg/day [267]	CS at 0.5 to 1 mg/kg [267]	Dapsone, at 50–150 mg/day for adults, 0.5 to 2 mg/kg/day for children [306]	GFD and dapsone [307]
Prognosis	The overall survival rates at 5 years were estimated at 92.9 [99]	The overall survival rates at 5 years were estimated at 96.7 [99]	The 5 years following mortality rates were estimated at 62 [99]	SMR: 1.9–7.2 [171]	Blindness, difficulty speaking and breathing [272]	Favorable prognosis	Favorable prognosis	Favorable prognosis	Favorable prognosis	Favorable prognosis with GFD [285]

This table presents the characteristics and progress in clinical management of various subtypes of AIBD

*Abbreviation*: *Dsg1* Desmoglein1, *Dsg3* Desmoglein3, *EC* extracellular, *PF* Pemphigus foliaceous, *PV* Pemphigus vulgaris, *PNP* Paraneoplastic pemphigus, *BP* Bullous pemphigoid, *NC* Noncollagenous, *LAD-1* Linear IgA bullous dermatosis autoantigen 1, *LABD* Linear IgA bullous dermatosis, *MMP* Mucous membrane pemphigoid, *OCP* Ocular cicatricial pemphigoid, *EBA* Epidermolysis bullosa acquisita, *DH* Dermatitis herpetiformis, *pmp/y* per million population per year, *BMZ* Basement membrane zone, *CS* Corticosteroids, *SMR* Standardized mortality ratio, *GFD* Gluten-free diet

**Table 2:** Advances in autoimmune bullous diseases in the last 5 years

Classification		Latest Research Advances	
		Immune Mechanisms	Targeted Therapy
Pemphigus disease	Pemphigus vulgaris	1. ELSs in pemphigus lesions might act as a niche, supporting in situ B cell differentiation and clonal expansion [61] 2. T follicular helper-like CD4(+) T cells in pemphigus lesions promoted local autoantibody production, resulting in the formation and recurrence of lesions [127] 3. Desmoglein-Specific B-Cell-Targeted Single-cell analysis revealing unique gene regulation in patients with pemphigus [62] 4. Skin TLSs are associated with the persistence of chronically recurrent blisters in patients with pemphigus, and the microenvironmental network involving CXCL13 + CD4 + T cells and Tregs within these structures plays an important role in CXCL13 production 5. Pemphigus autoantibody-induced Dsg3 epitope-specific signalling which is involved in pathogenic events such as Dsg3 depletion [93] 6. IL-1α signaling pathway, myeloid APCs, inflammatory CD8 + resident memory T cells, and dysfunctional CD4 + regulatory T cells are involved in the pathogenesis of PV [64] 7. The enhanced cell communication between stromal cells and immune cells like B cells and macrophages/dendritic cells was also identified in PV lesions [63] 8. Interaction between CCL19 + Inflammatory Keratinocytes and CCR7 + Dendritic Cells and B Cells in Pemphigus [67] 9. Chemokine-receptor mapping uncovers cell-type-specific signaling programs involved in the recruitment of T/B cells within pemphigus lesions [65]	RTX [112], OFA [116], BTKi, BAFF inhibitor [105], the FcRn antagonist [314], CAART [126]
	Pemphigus foliaceus		RTX [112], OFA [116], the FcRn antagonist [314], IL-17 blockers, TNF-α inhibitors, JAKi
Paraneoplastic Pemphigus		1. Non-Hodgkin lymphoma and lichenoid/interface dermatitis remained associated with the prognosis of PNP patients [156]	RTX, BTKi, alemtuzumab, daclizumab, tocilizumab [315]
Pemphigoid	Bullous pemphigoid	1. IL-17 A is functionally relevant and a potential therapeutic target in bullous pemphigoid [194] 2. Epitope-spreading phenomenon in pemphigoid [316] 3. The enhanced cell communication between stromal cells and immune cells like B cells and macrophages/dendritic cells was also identified in BP lesions [63] 4. Chemokine receptor mapping revealed the potential roles of macrophages and mast cells in recruiting pathogenic immune cells and underlying mechanisms within BP lesions [317] 5. Eosinophil extracellular traps drive T follicular helper cell differentiation via VIRMA-dependent MAF stabilization in bullous pemphigoid [318] 6. IL-24 is related to bullous pemphigoid severity [67, 319]	RTX, BAFF inhibitor, the FcRn antagonist, dupilumab, Omalizumab, JAKi [283, 316]
	MMP		RTX, TNF-α inhibitors, JAKi [272]
	EBA		RTX [266]
	Anti-p200 pemphigoid		RTX [270], JAKi
IgA-related AIBD	IgA pemphigus	NA	RTX, TNF-α inhibitors [310]
	LABD	NA	RTX, TNF-α inhibitors, Omalizumab [306]
	DH	NA	RTX [285]

This table presents the advances in AIBD in the last 5 years

*Abbreviation*: *PF* Pemphigus foliaceous, *PV* Pemphigus vulgaris, *ELS* Ectopic lymphoid-like structures, *TLS* Tertiary lymphoid structures, *APC* Antigen-presenting cells, *PNP* Paraneoplastic pemphigus, *BP* Bullous pemphigoid, *VIRMA* Vir-like mA methyltransferase-associated protein, *MAF* Musculoaponeurotic fibrosarcoma, *LABD* Linear IgA bullous dermatosis, *MMP* Mucous membrane pemphigoid, *EBA* Epidermolysis bullosa acquisita, *AIBD* Autoimmune bullous diseases, *DH* Dermatitis herpetiformis, *RTX* Rituximab, *OFA* Ofatumumab, *JAKi* Janus kinase inhibitors, *BTKi* Bruton's tyrosine kinase inhibitor, *CAART* Chimeric autoantibody receptor T cells

To some extent, the evolving understanding of AIBD has transformed it from a purely skin disorder to a complex systemic disorder involving multiple immune disorders. While DIF remains the diagnostic cornerstone, emerging molecular technologies have refined disease classification through autoantigen identification [315, 320]. Notably, the recognition that certain AIBD subtypes exhibit immune-mediated inflammatory disease (IMID) characteristics (e.g., Th2-mediated inflammation in bullous pemphigoid [317, 318]) has provided critical insights into their associations with comorbidities like inflammatory bowel disease and neurological disorders [321, 322]. However, not all AIBDs fit the IMID paradigm (such as MMP), emphasizing the need for mechanism-based disease stratification [323].

Current challenges persist in both diagnosis and treatment. Conventional immunoassays often fail to address antigenic complexity and intermolecular epitope spreading [316, 324–327], necessitating development of multivariant detection platforms [328, 329]. While targeted therapies like rituximab have revolutionized pemphigus management, recent clinical trial setbacks (e.g., BAFF inhibitors [314]) underscore the importance of rigorous trial design and deeper pathophysiological understanding. The dissociation between autoantibody titers and clinical severity in certain subtypes suggests additional inflammatory mechanisms beyond humoral immunity, supported by single-cell and proteomic studies revealing eosinophil extracellular traps and cytokine networks [319].

Future progress demands integration of multi-omics approaches to decode shared molecular pathways across AIBDs and IMIDs. This paradigm shifts from organ-based to mechanism-driven classification could enable: (1) Identification of predictive biomarkers for disease progression and treatment response; (2) Development of personalized therapies targeting core inflammatory pathways (e.g., JAK-STAT, IL-4/IL-13 signaling); and (3) Redefinition of disease boundaries through cross-disciplinary analysis of chronic inflammatory conditions. Ultimately, bridging the gap between bench discoveries and bedside applications will require sustained collaboration between clinicians and translational researchers, leveraging advanced technologies to overcome current diagnostic limitations and therapeutic challenges in AIBD management.

## Data Availability

Not applicable.

## Abbreviations

AIBD: Autoimmune Bullous Diseases
BMZ: Basement membrane
DIF: Direct immunofluorescence
ELISA: Enzyme-linked immunosorbent assay
Dsg: Desmoglein
PV: Pemphigus vulgaris
PF: Pemphigus foliaceus
PH: Pemphigus herpetiformis
PNP: Paraneoplastic pemphigus
PAMS: Paraneoplastic autoimmune multiorgan syndrome
Bregs: Regulatory B cells
EC: Extracellular
PDAI: Pemphigus disease area index
MMF: Mycophenolate mofetil
AZA: Azathioprine
MPA: Mycophenolic acid
MTX: Methotrexate
CTX: Cyclophosphamide
CsA: Cyclosporine A
IVIG: Intravenous immunoglobulin
RTX: Rituximab
CDC: Complement-dependent cytotoxicity
ADCC: Antibody-dependent cell-mediated cytotoxicity
OFA: Ofatumumab
BTK: Bruton's tyrosine kinase
BAFF: B-cell activating factor
JAKi: Janus kinase inhibitor
CAART: Chimeric Autoantibody Receptor T Cells
MDC: Multidisciplinary care
ABSIS: Autoimmune bullous skin disorder intensity score
A2ML1: Alpha-2-macroglobulin-like protein 1
TNF-α: Tumor necrosis factor-alpha
EVPL: Envoplakin
PPL: Periplakin
IIF: Indirect immunofluorescence
BP: Bullous pemphigoid
MMP: Mucous membrane pemphigoid
EBA: Epidermolysis bullosa acquisita
LABD: Linear IgA bullous dermatosis
UV: Ultraviolet
NSAIDs: Non-steroidal anti-inflammatory drugs
DPP-4: Dipeptidyl peptidse-4
PD-1: Programmed cell death protein 1
Tfh: T follicular helper
NC: Non-collagenous
SCC: Squamous carcinoma cells
IC: Intracellular
OCP: Ocular cicatricial pemphigoid
SLE: Systemic lupus erythematosus
BPDAI: BP disease activity index
ADRs: Adverse drug reactions
ENT: Ear, nose, and throat
IEN: Intraepidermal neutrophilic
SPD: Subcorneal pustular dermatosis
DH: Dermatitis herpetiformis
TG2/tTG: Tissue transglutaminase
TG3/eTG: Epidermal transglutaminase
GFD: Gluten-free diet
IMID: Immune-mediated inflammatory disease
